# The Impact of COVID-19 Infection on Abdominal Aortic Aneurysms: Mechanisms and Clinical Implications

**DOI:** 10.1155/2024/7288798

**Published:** 2024-07-29

**Authors:** Zenghan Cao, Jianhang Gao, Jianqiang Wu, Yuehong Zheng

**Affiliations:** ^1^ Department of Vascular Surgery Peking Union Medical College Hospital Chinese Academy of Medical Sciences and Peking Union Medical College, Beijing 100730, China; ^2^ Institute of Clinical Medicine National Infrastructure for Translational Medicine Peking Union Medical College Hospital Chinese Academy of Medical Sciences and Peking Union Medical College, Beijing 100730, China; ^3^ State Key Laboratory of Complex Severe and Rare Disease Peking Union Medical College Hospital, Beijing 100730, China

**Keywords:** abdominal aortic aneurysm (AAA), COVID-19, hypercoagulation, immune system, renin-angiotensin-aldosterone system (RAAS)

## Abstract

**Background:** The COVID-19 virus not only has significant pathogenicity but also influences the progression of many diseases, altering patient prognosis. Cardiovascular diseases, particularly aortic aneurysms, are among the most life-threatening conditions.

**Main Idea:** COVID-19 infection is reported to accelerate the progression of abdominal aortic aneurysms (AAAs) and increase the risk of rupture; however, a comprehensive understanding of the underlying mechanisms remains elusive. This article primarily reviews the relevant foundational research, focusing on disruptions in the renin-angiotensin-aldosterone system (RAAS), immune system activation, and coagulation disorders. Furthermore, we summarize related clinical research, including the epidemiology of aortic aneurysms during the pandemic and specific case studies.

**Conclusion:** COVID-19 infection can influence the onset and progression of aortic aneurysms by affecting the RAAS, triggering inflammation and immune dysregulation in the arterial wall, and inducing a hypercoagulation state. It is crucial to comprehensively understand the impact of pandemic viral infections on aortic diseases at the foundational and clinical levels, thereby identifying potential preventative or therapeutic approaches and preparing for potential future outbreaks.

## 1. Introduction

COVID-19 virus has been implicated in various diseases, particularly in cardiovascular disease. It does accelerate disease progression which in turn affects patient prognosis. Several reviews have described the relationship between novel coronavirus infection and prevalent cardiovascular diseases, such as myocardial injury, atrial fibrillation, pulmonary embolism, myocarditis, heart failure, and disseminated intravascular coagulation [1]. Abdominal aortic aneurysms, or AAAs, have an elevated risk of rupture leading to fatal outcomes particularly during infectious outbreaks, necessitating particular attention during periods of epidemic prevalence.

AAA represents a substantial clinical challenge due to the localized enlargement of the abdominal segment of the aorta, the reported incidence of which ranges from 1.2% to 4% among adults. This has been seen particularly in the elderly population, and the fatalities are increasing mainly as a result of ruptures [2]. The pathogenesis of AAA is rooted in the molecular degradation of structural proteins in the aortic wall, a process that compromises wall integrity and predisposes the wall to eventual rupture [3].

In light of the COVID-19 outbreak, numerous healthcare institutions have documented instances of abrupt aneurysm expansion, even leading to rupture, in patients with AAAs. The rupture rate of AAAs has shown a discernible surge during this period [4, 5]. The possibility that the novel coronavirus acts as a direct stimulant of AAA development and progression cannot be disregarded. Although the World Health Organization has declared the current COVID-19 pandemic to be over [6], we still face uncertainty surrounding the future. Should a new infectious outbreak occur, the lessons learnt from COVID-19 outbreak would be extremely beneficial particularly for diagnosis and treatment. Hence, a retrospective review of the impact of COVID-19 infection on the pathogenesis of AAA and its influence on clinical diagnostics and treatment is urgently needed.

The intricate interplay between COVID-19 infection and AAA has been underscored by the observed dysregulation of numerous pathways [7]. The inflammatory and coagulation responses triggered by infection invariably exert detrimental effects on the cardiovascular system. Substantial perturbations have been identified in the vascular bed of various tissues with prevalent thrombosis and microangiopathy [8]. Also, COVID-19 affects medium-to-large blood vessels [9].

Several studies have established correlations between the severity of COVID-19, comorbidities, and AAA, yet the majority are focused on singular pathways rather than offering a comprehensive review. Additionally, the synergistic effects of various dysregulations in accelerating disease progression have often been overlooked. On the other hand, there is a notable scarcity of literature summarizing the impact of COVID-19 infection on the clinical diagnosis and treatment of AAA. This article seeks to address this gap by summarizing the potential mechanisms by which COVID-19 influences the pathogenesis of AAA, as well as the challenges encountered in the diagnosis and treatment of AAA during the pandemic outbreak. Hopefully, the insights gained will be instructive in shaping the response to potential future epidemic events.

## 2. Methods

Electronic searches for studies were conducted across PubMed, MEDLINE, Embase, and Cochrane databases up to December 22, 2023, employing search terms such as “SARS-CoV-2”, “COVID-19”, “2019 novel coronavirus”, “SARS2”, “abdominal aortic aneurysm”, “thrombosis”, and “vascular”. There were no restrictions on the type of articles included, encompassing original articles, reviews, letters, and conference abstracts. Both clinical and basic research studies were considered for inclusion.

## 3. Results

### 3.1. Clinical Evidence Supporting the Effect of COVID-19 on AAA

Complex aortic conditions are often associated with inflammatory conditions such as autoimmune and infectious diseases due to histopathological modifications that result in weakening of the aortic wall [10]. Several infectious agents have been identified as potential inducers of aortic lesions, which may result in aneurysms that can eventually cause rupture or dissection [11, 12].

Several surgeons, through imaging and clinical observation, have postulated that the virus may affect the aorta. They noted a thickening of perivascular tissue, similar to what is observed in inflammatory aortopathies, during surgical interventions. A study conducted by Vlachopoulos et al. utilized ^18^F-FDG-PET/CT imaging to assess aortic inflammation and revealed an increase in the early COVID-19 infection phase [13]. Akgul et al. reported the treatment of aortic type A dissection in a patient positive for COVID-19 [14]. Some case reports have included instances of AAA in COVID-19 patients, highlighting postoperative complications such as suture line bleeding, often attributed to a loss of elasticity and strength in the aortic wall, which is a common occurrence in inflammatory aortopathy [10, 11]. It has been hypothesized that COVID-19 infection may have contributed to the observed aortic wall inflammation and ensuing surgical complications [15]. Manenti et al. provided a comprehensive overview of the pathophysiological mechanisms underlying aortitis in patients with COVID-19 [16]. They proposed that the pathology of vasculitis could affect large arteries, including the aorta.

### 3.2. Pathophysiological Mechanisms of COVID-19 Influencing AAA

The interplay between COVID-19 and AAA has been substantiated by a growing body of foundational scientific evidence [15]. The SARS-CoV-2 virus capitalizes on Angiotensin-Converting Enzyme (ACE) 2 (ACE2) to gain access to target cells, thereby initiating infection. The vascular wall, which comprises a variety of cell types, highly expresses ACE2, suggesting that it is a direct target for preventing COVID-19 invasion. The ensuing weakening of the vascular wall due to viral infection could contribute to the genesis and progression of aneurysms [16].

**Figure 1:**
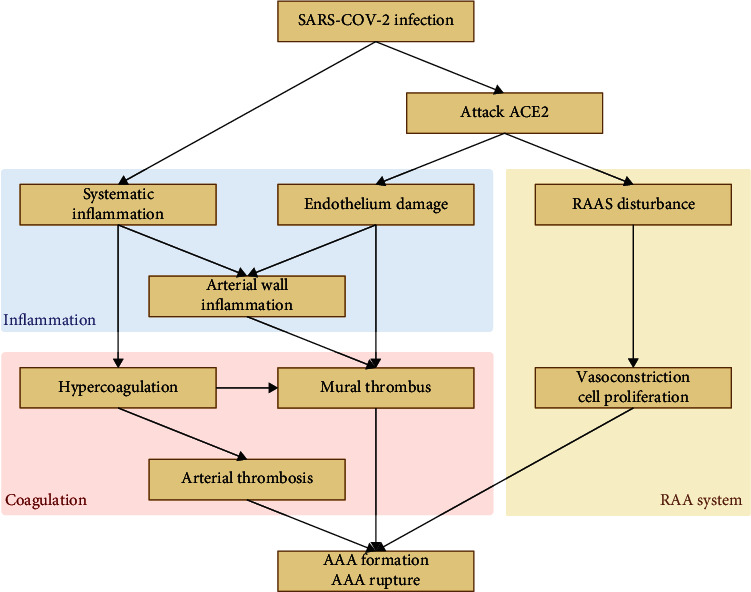
The RAAS, immune system, and coagulation system contribute to the interplay between COVID-19 and AAA.

The impact of COVID-19 on AAA is multifaceted, precipitating disruptions in the immune system, the renin-angiotensin-aldosterone system (RAAS), and thrombotic equilibrium (Figure 1). Foremost, COVID-19 interrupts ACE2 expression by fostering its cleavage, thereby diminishing the protective effect of ACE2 on endothelial cells (ECs) and other organs. This action triggers the activation of the RAAS, escalating innate immune stimulation [17]. Second, systemic inflammation, akin to a cytokine storm, macrophage activation syndrome, and immune exhaustion, has been documented in COVID-19 patients. Observations of endothelial damage, reminiscent of vasculitis, highlight the importance of pathological autoimmune responses in antiviral immunity [18]. Both direct viral effects and perivascular inflammation could contribute to these phenomena [15]. Last, the cytokine storm is also linked to disturbances in the pro- and antithrombotic balance, culminating in a state of hypercoagulability. This may result in thrombosis within the microvasculature and the aneurysm lumen. Intraluminal thrombus (ILT) formation may isolate blood flow from the arterial wall, resulting in a hypoxic state within the arterial wall and progressive weakening of the wall, ultimately contributing to the formation and potential rupture of an aneurysm [19].

#### 3.2.1. RAAS Disturbance and ACE2-Mediated Inflammation

**Figure 2:**
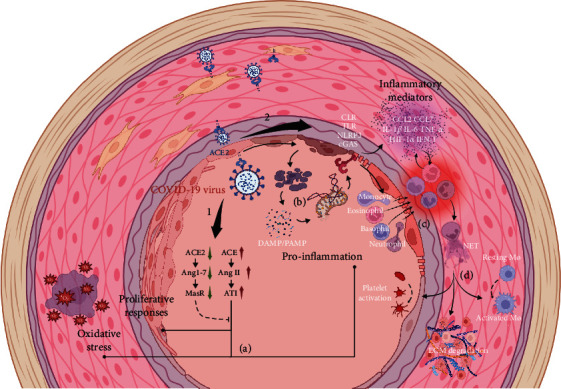
The role of the RAAS and immune system in COVID-19-AAA pathology. (a) The ACE-Ang II-AT1R axis is activated to foster inflammatory, oxidative stress, and proliferative reactions. (b) Necroptosis-related proteins release DAMPs and PAMPs to elevate a wide array of innate immune regulators. (c) Activated ECs increase endothelial permeability and promote the recruitment of inflammatory cells. (d) NETs promote platelet adhesion, macrophage activation, and ECM degradation. Abbreviations: ACE, Angiotensin-Converting Enzyme; Ang, angiotensin; AT1R, Angiotensin Type 1 Receptor; MasR, Mas receptor; DAMP, damage-associated molecular pattern; PAMP, pathogen-associated molecular pattern; TLR, Toll-like receptor; CLR, C-type lectin receptor; IL, interleukin; TNF, tumor necrosis factor; HIF, Hypoxia-Inducible Factor; ECM, extracellular matrix.

In the context of vascular health and function, the RAAS plays a pivotal role and is implicated in the pathogenesis of COVID-19. First, the destructive axis involving ACE, Angiotensin II (Ang II), and Angiotensin Type 1 Receptor (AT1R) is known to elicit proinflammatory and proliferative reactions in target cells, inducing oxidative stress and vasoconstriction [20]. Conversely, in the ACE2-Ang 1-7-Mas receptor (MasR) axis, a protective agent exerts counteractive effects [21]. Under normal physiological conditions, ACE2 counterbalances the RAAS by transferring Angiotensin I and II to Angiotensin 1–9 and 1–7, respectively [22], thereby protecting the aorta from inflammatory pathology [21] (Figure 2).

A reduction in ACE2 expression in AAA patients has been documented. In murine AAA models, both plasma and aortic tissue show downregulation of ACE2 and upregulation of Ang II [23]. Furthermore, ACE2 expression in various aortic vascular wall cells (e.g., ECs, smooth muscle cells, pericytes, fibroblasts, and certain immune cells) is markedly lower in AAA patients than in controls [21]. Concurrently, elevated plasma Ang II levels have been detected in COVID-19-infected individuals [24]. For animal models, while whole-body ACE2 deficiency may not contribute to the progression and severity of elastase-induced AAA [25], it is essential to note that the ACE2 axis plays a crucial role in the determination of Ang II–driven AAA formation [26]. Administration of Ang 1–7, the Ang II degradation product, can mitigate AAA [27, 28], while inhibition of ACE2-related receptors exacerbates AAA [29, 30]. Consequently, we hypothesize that a reduction in ACE2 expression and an imbalance in Ang II/Ang 1–7 due to COVID-19 infection could accelerate AAA progression [21].

In addition to affecting the RAAS, coronavirus disrupts the immune system via the binding of its spike protein to ACE2. This interaction is postulated to trigger a cascade of inflammatory mediators (e.g., C-C Motif Chemokine Ligand (CCL)–2, interleukin (IL)-1*β*, IL-6, and tumor necrosis factor (TNF)–*α*) in ACE2-positive cells. ACE2-positive arterial vascular cells are enriched in CCL2-CXCL12 relative to ACE2-negative cells in diseased patients, as evidenced by single-cell RNA analysis [31]. Given that virtually all constitutive vascular cells express ACE2, COVID-19 may potentiate AAA pathogenesis by enhancing aortic leukocyte recruitment and/or augmenting aortic wall inflammation [32]. Certain ACE2-producing cells may also secrete ligands that bind to receptors on ACE2-negative cells, such as macrophages and neutrophils; promote the release of proinflammatory factors, including CCL2, CCL7, Hypoxia-Inducible Factor (HIF)–1a, and Type 1 Interferon (IFN-1); and exacerbate the inflammatory response during AAA formation [33, 34].

#### 3.2.2. Immune Dysregulation

As an invasive microorganism, the novel coronavirus naturally triggers a nonspecific immune response, such as the activation of immune cells such as macrophages, monocytes, T lymphocytes, and mast cells. This can lead to an inflammatory storm characterized by the overproduction of cytokines and chemokines. Concurrently, viruses can also activate the complement system, forming a feedback loop in which the complement, immune, and coagulation systems mutually influence each other. Persistent inflammation can encourage arterial wall cell apoptosis, phenotypic switching, and the production of proteases, which induce extracellular matrix degradation. In terms of aortic tissue, COVID-19 infection can trigger inflammation in the aortic wall through specific pathways (Figure 2).

As a vasculopathic disease, COVID-19 primarily targets ECs, which are located in the aortic intima and are pivotal for maintaining aortic homeostasis. Numerous studies have implicated EC dysfunction as a significant determinant of AAA [35]. Viral assault on these cells results in endotheliitis. The aorta functions as an immune organ during viral infection according to the criterion of facilitating immune cell maturation, differentiation, and activation [36].

As previously discussed, viral infection induces macro- and microvascular endothelial dysfunction and damage by bridging ACE2 and the COVID-19 spike protein. Necroptosis, a form of cell death, can occur through the release of damage-associated molecular patterns (DAMPs) and pathogen-associated molecular patterns (PAMPs), which are detected by specialized receptors such as Toll-like receptors (TLRs) and C-type lectin receptors. These receptors elevate a wide array of innate immune regulators in various types of ECs [37]. Activated ECs also increase endothelial permeability and attract immune cells, thereby promoting the recruitment of inflammatory cells to damaged tissues [15, 36, 38]. Additionally, coronaviruses may stimulate the inflammatory response in the endothelium through the cGAS-STING-GMP-IFN pathway. Mitochondrial DNA released due to viral infection is detected by receptors (TLR-9, NOD-Like Receptor Thermal Protein Domain Associated Protein 3 (NLRP3), and cyclic GMP-AMP synthase (cGAS)) that typically identify viral or bacterial DNA. These receptors may stimulate the expression of various cytokine genes, including interferons, ILs, and TNF-alpha, all of which play significant roles in aortic aneurysm disease [15, 35, 39, 40].

Among these cytokines, IFN is deemed crucial. Numerous studies have reported an increase in the IFN level in COVID-19-infected cells. Furthermore, IFN-1 is a cytokine involved in AAA pathogenesis that functions by binding to its receptor and interferon regulatory factors. In addition, IFN-1 and several other cytokines (IL-13, apelin, Ang II, hypoxia, and resveratrol) influence the ACE2-Ang 1-7-MASR axis by increasing ACE2 expression and activity [41, 42]. Most Type 1 INFs initiate intracellular signaling cascades and Type 1 IFN–regulated gene expression through binding to the Type I IFN heterodimer receptor subunit (IFNAR) 1/IFNAR2 [43]. Shoji et al. revealed that IFNAR1 expression was upregulated in clinical and experimental AAAs. A deficiency in IFNAR1 expression led to a reduced incidence and progression of experimental AAAs [44]. Furthermore, histological analyses revealed that IFNAR1 deficiency resulted in relative preservation of the vascular wall structure, including the cells (smooth muscle cells and macrophages) and extracellular matrix (medial elastin), reduced immune cell recruitment, and neoangiogenesis. These findings suggest a potential mediating role for IFN-IFNAR1 in AAA pathogenesis [44].

Additional microenvironmental dysregulation during COVID-19 infection might contribute to the pathogenesis of aneurysms. The immune response activated by EC activation or necroptosis can also cause significant production of neutrophil extracellular traps (NETs), which contribute to local tissue damage [45]. NETs, net-like complexes comprising chromatin DNA, neutrophil granule proteins, and histones, are released into the extracellular space for immune function [46]. NETs are thought to be nonspecific immune elements that trap pathogens to prevent their spread. The reticulate structure concentrates antimicrobial factors to eradicate pathogens [47]. Elevated levels of NET parameters, such as cell-free DNA, MPO-DNA complexes, and citrullinated histone H3, were detected in the blood serum of COVID-19 patients [47]. Initial vascular damage and subsequent organ dysfunction are associated with excessive NET formation in patients with severe COVID-19 [47]. NETs may also contribute to the inflammatory pathogenesis of noninfectious diseases, particularly cardiovascular diseases [48]. Several basic experiments have provided evidence that NETs and other biological activities of neutrophils play crucial roles in AAA pathogenesis [47, 49–51]. A deficiency in plasma neutrophils inhibits AAA development in experimental animals [50]. NETs can also stimulate receptors on the surface of macrophages, such as NLRP3, thereby upregulating the expression of IL-1*β* and IL-18 in arterial tissue. These inflammatory factors can, in turn, further promote the production of NETs, forming a positive feedback loop [52]. Anti-NET therapies for AAA, such as DNase I and chloro-amidines, have been applied to test their effectiveness. These inhibitory mediators, which suppress NETosis (inflammatory cell death modality of neutrophils), slow aneurysm formation in experimental AAA mouse models [49, 51].

In addition to NETosis, the defense mechanism of neutrophils includes phagocytosis and degranulation [53]. The degranulation of azurophilic granules releases the protease neutrophil elastase [54]. The primary function of neutrophil elastase is to degrade components of the extracellular matrix, accelerating proinflammatory cells (granulocytes and macrophages) to pass through tissue and reach sites of inflammation [54]. It was recently suggested that neutrophils are implicated in the devastating immunoinflammation responsible for secondary COVID-19-related pulmonary inflammation [55]. In AAA, functional imaging of AAA patients suggested that inflammation in the aortic wall contributes to its degradation [56]. The progressive disease state is further exacerbated by the activity of elastase, which precipitates elastin for degradation and aneurysm formation. The progressive disease state is further exacerbated by the activity of elastase, which precipitates elastin degradation and aneurysm formation. A novel positron emission tomography tracer, GW457427, which is selective and specific to neutrophil elastase, has been used in human in vivo studies. These investigations demonstrated elevated activity of neutrophil elastase within the vascular wall of human AAA tissue compared with that of a healthy aorta [54]. Therefore, in the context of COVID-19 infection, an upregulated neutrophil elastase response may significantly contribute to inflammation of the aortic wall during the pathogenesis of AAA.

#### 3.2.3. Hypercoagulation Status

**Figure 3:**
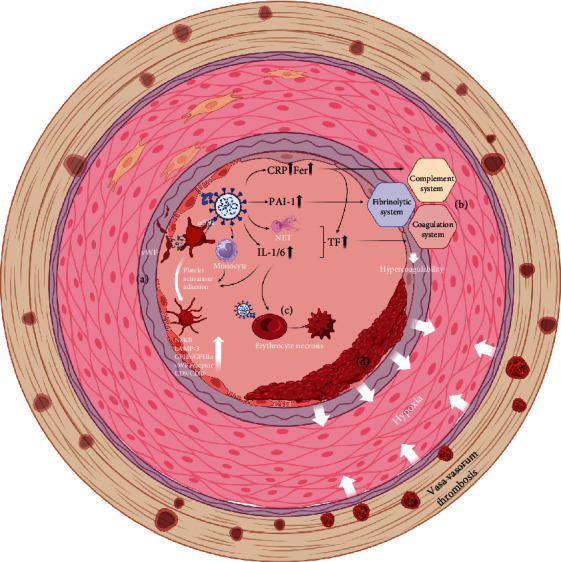
Pathology of the coagulation system in patients with COVID-19-AAA. (a) Viruses can activate downstream pathways and induce platelet adhesion by activating inflammatory cells, releasing cytokines, or interacting with platelet membrane proteins directly. (b) Systemic inflammatory mediators may serve as pathophysiological links between inflammation and thrombotic events by disrupting the equilibrium among the complement system, fibrinolysis, and coagulation cascades. (c) COVID-19 can directly interact with red blood cells or indirectly alter cell structure to promote thrombosis. (d) An intraluminal thrombus can exacerbate parietal hypoxia of aneurysm walls. Abbreviations: vWF, von Willebrand factor; LAMP, lysosomal-associated membrane protein; NET, neutrophil extracellular trap; CRP, C-reactive protein; SF, serum ferritin; PAI-1, Plasminogen Activator Inhibitor 1; TF, tissue factor.

In approximately 75% of AAAs, the presence of an ILT is discernible. ILT is typically characterized by concomitant destruction of the nearby endothelium and abundant inclusion of macrophages, neutrophils, erythrocytes, and platelets [57] (Figure 3). Research has suggested that ILTs engender a deleterious microenvironment that impedes appropriate oxygen transport within the aortic wall and exacerbates inflammation. Hence, the cumulative impact of ILT on AAA tends to be pathogenic rather than protective [35, 58].

In the context of COVID-19 infection, it is plausible that the adaptive cell-mediated immune response may become activated. This could lead to aberrant antibody and autoantibody production, thereby exacerbating the hypercoagulable state and disrupting the neutrophil–platelet axis. These alterations can culminate in fatal thrombotic events [59, 60]. Certain systemic inflammatory factors can also link inflammation and thrombotic events. For instance, C-reactive protein can simultaneously affect the balance of the complement and fibrinolysis systems and promote platelet adhesion and the expression of tissue factor, all of which can lead to a hypercoagulable state [61]. Ferritin, another example, can promote the dysfunction of mitochondria within platelets, thereby promoting inflammation and thrombosis [62]. In addition to cytokines, many immune cells can also promote the transition from inflammation to thrombosis. For example, monocytes can interact with platelets, and neutrophils can produce NETs [63–65]. These inflammatory changes can all promote various parts of the coagulation system to exert their coagulation effects. Platelets are an integral part of the coagulation process. COVID-19 can interact with the platelet membrane protein integrin *α*5*β*1 or upregulate IL 1/6 to promote the activation of downstream pathways within platelets, such as the NF*κ*B pathway. Moreover, platelet activation markers, including Lysosomal-Associated Membrane Protein 3, the GPIIb/GPIIIa complex, von Willebrand factor (vWF) receptor units, CD9, and CD40, are upregulated, thereby inducing platelet adhesion and other coagulation-related processes [66, 67]. vWF, a multimeric glycoprotein with procoagulant properties, is a crucial factor in blood coagulation and is synthesized by ECs. The interplay between NF*κ*B2-mediated vWF transcription and the ADAMTS13-vWF axis could be pivotal in bridging immunity and thrombosis; hence, potential disruption of the hemostatic system could occur [64, 68].

In the context of COVID-19 patients, the observed impairment of fibrinolysis is a consequence of elevated Plasminogen Activator Inhibitor 1 (PAI-1) levels. This molecule, a member of the serine protease inhibitor superfamily, impedes the conversion of the precursor plasminogen to its active form, plasmin [69]. In addition, the activation of the complement system, imbalances in the fibrinolytic system, and activation of the IL-NET axis caused by COVID-19 infection can also upregulate tissue factor expression and promote the extrinsic coagulation pathway [70]. COVID-19 can also directly interact with red blood cells, either through contact with surface proteins on the surface of red cells or indirectly by altering the structure of red cells through elevated levels of IL family factors, leading to the nonprogrammed death of red cells and thus thrombosis [71].

In a state of heightened coagulation, associations can be drawn between the occurrence of vasa vasorum thrombosis and the expeditious augmentation of ILT. Through their firm adhesion to aneurysm walls, the latter exacerbates parietal hypoxia [59]. Consequently, oxidative stress escalates within the aortic wall, leading to the accumulation of inflammatory cells and the upregulation of elastases and metalloproteinases [16, 47, 59]. Notably, hypercoagulation, as a repercussion of COVID-19 infection, may heighten the risk for thrombosis. While the majority of observed cases pertained to venous thromboembolism, a notable proportion also manifested as arterial thrombosis, encompassing acute lower limb ischemia and postoperative vascular prosthesis, particularly in patients fitted with a vascular prosthesis [12, 72, 73]. Giacomelli et al. presented the inaugural case of acute thrombosis in a prosthetic aortic graft observed in a COVID-19-positive patient devoid of any anatomical predispositions for thrombosis [72].

### 3.3. Impact of COVID-19 on the Diagnosis and Treatment of AAA

In addition to fundamental research on the impact of COVID-19 on AAA, there are also numerous reports summarizing changes in AAA diagnosis and treatment during the pandemic. To date, we have experienced numerous outbreaks of COVID-19 infection worldwide, and newly emerged variants are constantly posing challenges for both public health in general and for AAA, which is a disease that relies on screening and surveillance. There are concerns that AAAs may not be diagnosed in a timely manner or managed closely, increasing the risk of serious complications and potentially affecting patient prognosis.

There is ample evidence of the impact of COVID-19 on cardiovascular disease incidence. Early in mid-2020, when outbreaks were concentrated in several regions around the globe, reports about the impact of COVID-19 on common cardiovascular emergencies, including ST-elevation myocardial infarction, stroke, and AAA, revealed delayed treatment and decreased transfer to a higher level of care centers [74]. Infection of COVID-19 may also contribute to the accelerating growth of AAA, as demonstrated by retrospective clinical studies and animal experiments. Xu et al. found that patients who reported a previous COVID-19 infection were 9.7 times as likely to have rapid AAA growth. At the same time, administration of spike Protein 1, a protein the COVID-19 virus uses to enter cells, to standard mice and transgenic mice for the AAA model could lead to significant growth of aneurysms in both types of mice when infected with the COVID-19 virus [75]. Bozzani et al. [8, 76] reported nine cases of acute thrombosis occurring during or immediately after COVID-19 infection; 6 patients underwent thrombectomy, while in another series, 6 acute arterial and 32 deep vein thromboses resulted in 10 deaths and 1 amputation despite aggressive therapy, highlighting the importance of close follow-up and anticoagulation in similar cases. They also reported four cases of COVID-19-related ruptured AAA; two patients underwent open surgery repair, while the other patient underwent endovascular repair [77]. The increased risk of ruptured AAAs during COVID-19 infection may be related to multiple factors, including the use of steroids and the risk of severe vascular disease during the COVID-19 pandemic. Regional data confirmed a decrease in elective operations and an increase in emergency operations involving vascular surgeries [78]. From the perspective of medical service providers, management and surveillance are estimated to be insufficient during the pandemic period, as indicated by increased rates of failure to control LDL levels in AAA or peripheral artery disease patients [79]. Chen et al. reported the possibility of diagnosing heterogeneous vascular disease by CT scanning during the pandemic, and close clinical follow-up and surveillance are suggested [80]. From the patients' perspective, this was also reflected in a survey of the willingness of patients to attend AAA surveillance during the pandemic period, as shown by Selway et al., who suggested that such programmes may be affected by a lack of participation from patients due to subjective or objective factors [81].

Sullivan et al. conducted research on the COVID-19 pandemic and vascular diseases using data from the Society for Vascular Surgery Vascular Quality Initiative [82]. Several notable issues related to the management and prognosis of vascular diseases, including AAA, have been identified. Concerns about possible delays in diagnosing and treating vascular diseases due to COVID-19 infection have increased, and an increase in mortality has been associated with a positive COVID-19 test, although these studies failed to prove that a delay in the procedure could lead to increased mortality. Provided that the restriction of elective or emergent procedure performance was limited, they concluded that the ratio of elective to nonelective surgeries was not significantly different from the historical level. However, these conclusions were challenged by the findings of other studies, which argue that the pandemic could hinder patients from screening and acknowledging their health status and that standard guidelines should be followed even under such challenges [83]. Specifically, single-center data revealed higher rates of ruptured AAAs during the pandemic period, as the emergency operation rate increased from 6% to 11.2%, potentially because of the lack of screening for patients with possible manifestations and postponed follow-up for small AAAs [84].

Management of AAAs, which may turn lethal, indeed raises serious challenges during the COVID-19 pandemic and similar scenarios. This was reflected in the longer waiting list for AAA surgery reported by Ramsay et al., who called for scheduled surgeries and closer follow-up and prepared for resolving a possible backlog of patients [85]. Kim et al. utilized a discrete event simulation model to investigate the impact of service disruption on AAA outcomes [86] and proposed that a delay in initial screening for AAA of up to 2 years could lead to little change in outcome, while a lack of surveillance for AAA patients could cause additional concerns. They proposed that recovery from AAA surveillance should be prioritized and that screening for AAA should also be encouraged. Innovations in the management of AAAs, such as telemedicine, which can provide patients with remote services [87], and novel clinical indicators, such as the probabilistic rupture risk index, were also reported by Kubicek et al. [88].

Treatment of AAA has raised another question, as a lack of screening and surveillance has added challenges to timely intervention, and a shortage of medical personnel and delayed services have occurred during pandemics. There is clearly a trade-off between treating patients with potential emergencies and saving medical resources [89]. Faggioli, Chakfé, and Imray proposed a checklist of elective aneurysm surgeries in the COVID-19 era, stressing the risk-return trade-off for individual patients and the necessity of balancing resources for COVID-19 patients. The authors also discussed the impact of psychological burdens on patients and the increased pressure of training surgical residents [90]. McGuinness et al. developed a decision tree for decision-making regarding immediate repair of AAAs, taking into account COVID-19 incidence and mortality, aneurysm rupture, and operative mortality, suggesting that more radical intervention is needed for younger patients and larger aneurysms [91]. A more systematic recommendation for intervention in AAA was proposed by Gwilym et al., who reported that deferral was preferred over open or endovascular repair of AAA at 3 months, while open repair was preferred in patients under 65 years old and aneurysms > 7 cm in diameter. The authors also noted the necessity of considering the risk of contracting COVID-19 transmission when evaluating whether patients should proceed to surgery [92].

AAA-related emergencies include ruptured AAAs and several severe complications. According to a multicenter retrospective study of ruptured AAA management during the pandemic period in China, there was a similar rate of endovascular repair and a similar short-term prognosis [93]. Multicenter data from Italy, which experienced a substantial impact of COVID-19 in 2020, demonstrated a decrease in the management of cardiovascular diseases [94]. Reported AAA emergencies combined with COVID-19 are not uncommon. Indications for surgery, anesthesia, thrombosis, and infection control were challenging, and proposed strategies or suggestions included endovascular surgery and early anticoagulation [5, 73, 95–97]. Endovascular surgery was preferred in most patients, which was consistent with the findings of other reports [98, 99]. Severe complications of AAA with COVID-19 infection have also been reported, such as in an elderly male patient who missed the opportunity to repair emergent Type III endoleaks of AAA and eventually died of AAA rupture [100].

## 4. Summary

New outbreaks may occur at any time, and research on novel infection mechanisms, and protective measures for severe complications such as AAA during different pandemic periods, necessitates drawing upon past experiences. We have delineated the potential mechanisms through which the novel coronavirus impacts the exacerbation of AAA. The virus capitalizes on the ACE2, diminishing its functionality and directly targeting specific cells. This action leads to instability within the RAAS and incites inflammation within the arterial wall. Simultaneously, systemic inflammation induces a hypercoagulable state. Therefore, the disturbance of RAAS, augmented by increased inflammation and coagulation, plays a significant role in the progressive development of AAA. Due to the infection may facilitate aneurysm development and rupture, AAA management during the COVID-19 pandemic is challenging. The clinical manifestations could be heterogeneous. With a better understanding and better control of the COVID-19 pandemic globally, we could look back and learn from the impacts of such pandemics on various diseases from both a physiobiological perspective and real-world management.

## Nomenclature

AAA: abdominal aortic aneurysm
ACE: Angiotensin-Converting Enzyme
Ang II: Angiotensin II
AT1R: Angiotensin Type 1 Receptor
CCL: C-C Motif Chemokine Ligand
DAMPs: damage-associated molecular patterns
ECs: endothelial cells
IFN: interferon
IFNAR: Type I IFN heterodimer receptor subunit
IL: interleukin
ILT: intraluminal thrombus
MasR: Mas receptor
NETs: neutrophil extracellular traps
PAMPs: pathogen-associated molecular patterns
RAA: renin-angiotensin-aldosterone
TLRs: Toll-like receptors
vWF: von Willebrand factor
